# Development and characterization of coaxial two-way switch for RF plasma discharge experiments

**DOI:** 10.1038/s41598-026-39452-0

**Published:** 2026-02-23

**Authors:** Raj Singh, Vishant Gahlaut, Vangalla Veera Babu, Uttam Goswami, Meenu Kaushik, L. Vijayakumar, Bhupender Sandher, Joydeep Ghosh

**Affiliations:** 1 Institute for Plasma Research, Gandhinagar, 382428 Gujarat India; 2 Department of Physical Sciences, Banasthali Vidyapith, Banasthali, 304022 India; 3 Homi Bhabha National Institute, Training School Complex, Anushaktinagar, Mumbai, 400094 India; 4 Department of Water Supply and Environmental Engineering, Arba Minch University, Arba Minch, Ethiopia; 5 Department of Electronics and Communication Engineering, Siddhartha Academy of Higher Education (Deemed to be University), Vijayawada, AP India

**Keywords:** RF switch, RF plasma discharge, Pre-ionization, Start-up, Wall conditioning, Ion cyclotron resonance heating, And SPDT, Energy science and technology, Engineering, Physics

## Abstract

A developed high-performance 3−1/8″ coaxial two-way RF switch tailored for RF plasma discharge experiments including pre-ionization and start-up; wall conditioning and Ion Cyclotron Resonance Heating has been presented (Bora et al. in Nucl Fusion 46:572–584, 2006; Wilson and Bonoli, in Phys Plasmas 22:021801, 2015). The proposed switch is configured as a coaxial two-way system, meticulously engineered to facilitate seamless transitions in coaxial connections between RF transmitters and antennas for RF power coupling to plasma and other applications and between RF transmitters and dummy loads as per the requirement, all accomplished with an impressively minimal changeover time. The foremost importance of this switch is that it can make two transmissions at a time in contrast to a normal SPDT (single pole double throw) switch where at a time only one connection is available and hence saves time and is economical. The other advantage of having two transmissions at a time is that the same RF load can be used for RF amplifier testing at various stages from 2 kW to 1.5 MW. The development process involves a thorough investigation into the unique requirements and challenges posed by the RF plasma discharge experiments, emphasizing the need for rapid and precise RF signal routing.

## Introduction

 A device used to direct high-frequency signals through transmission channels is called an RF (Radio Frequency) switch. Different combinations of RF and microwave switches allow for the creation of intricate matrices and automated systems for a wide range of applications. Single pole-multiple-throw (SPMT) switches provide a single input to multiple (three or more) output paths but only one output path at a time, while single-pole-double-throw (SPDT or 1:2) switches transport the signals from one input to one of the two output paths at a time. Double-Pole-Double-Throw (DPDT) or transfer switches can be used to bypass a component, switch between two inputs and two outputs, act as a drop-out switch, reverse a signal, or insert or remove a component from a signal path. There are two equally common and important categories for RF and microwave switches^1-3^.

1. Electromechanical Switch: The fundamental operation of electromechanical switches is based on the straightforward idea of electromagnetic induction. They use mechanical contacts as their switching mechanism.sss
2. Solid State Switch: A solid-state switch is a semiconductor-based electronic switching device (e.g. MOSFET, PIN diode). It works similarly to an electromechanical switch, with the exception that it lacks moving parts.

RF discharge experiments on Aditya and SST-1 are Ion Cyclotron Resonance Heating, RF pre-ionization, RF Wall conditioning in the superconducting coil environment, etc. RF power switches are required for these experiments to utilize the RF sources for various types of experiments at both the tokamaks – ADITYA and SST-1.

**Fig. 1:**
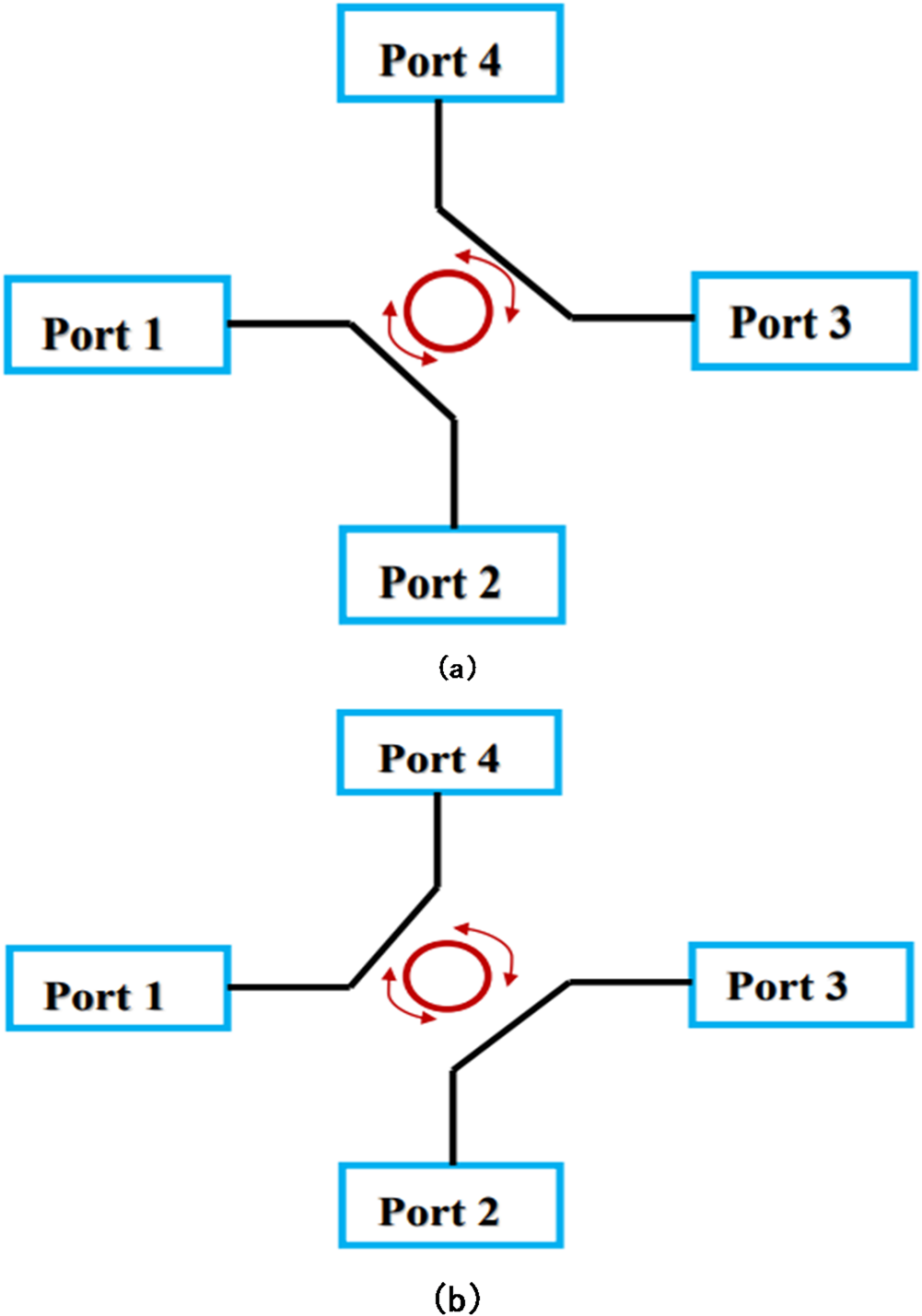
(**a**).Two-way coaxial RF Switch, Configuration-I. (**b**) Two-way coaxial RF Switch, Configuration-II.

One of the auxiliary heating methods used on ADITYA to increase the plasma temperature is the ion cyclotron resonance heating (ICRH) system. Depending on the applied toroidal magnetic field, it employs fundamental or second harmonic heating at frequencies between 20 and 40 MHz with an RF power of up to 500 kW. RF generator, a set of coaxial two-way switches that switch between the ADITYA tokamak machine and a 50-Ohm dummy load, a long 50-Ohm coaxial copper/Al transmission line from the RF generator to ADITYA hall, a matching network with phase shifters and shorted-stub tuners, and a vacuum interface section with a vacuum feed through and a shorted strip line plasma antenna are the components of the system^4^. The matching network matches the antenna impedance, which is of the order of a few ohms to the generator impedance of 50 ohms. As switch route power between dummy loads and ADITYA/SST-1, its design accuracy is very crucial as any deviation/deterioration in the design parameters will abnormally affect the power transmission. Two different configurations of the coaxial two-way RF switch have been shown in Fig. 1a, b. In one configuration the connection is between port 1 and 2 and port 3 and 4 while in another scenario the connection is between port 1 and 4 and port 2 and 3.

**Fig. 2:**
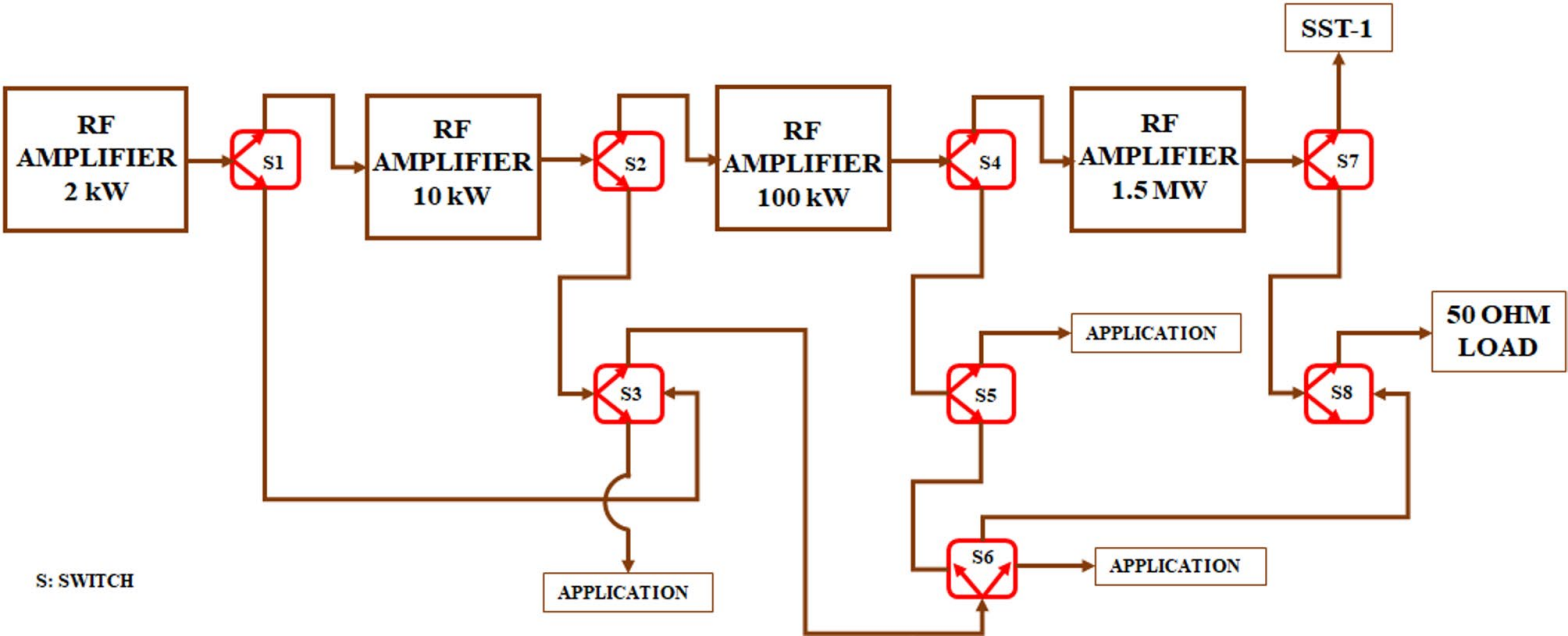
Schematic of a two-way coaxial switch connected in different configurations for various applications.

In SST-1 tokamak, RF discharges are used for RF pre-ionization, Ion Cyclotron Resonance Heating, and Wall conditioning^5^. One RF power amplifier chain has been installed in SST-1 with 2 kW, 10 kW, 100 kW and 1.5 MW power output at various stages. 10 kW output is through 3 1/8″, 100 kW output is through 6 1/8″ and 1.5 MW is through 9 1/8″ line. The power carrying capacity of the rigid coaxial transmission line depends upon voltage breakdown between inner and outer conductor which in turn depends upon its size. These various power levels are being used for various purposes and various stages of experiments. The developed 3 1/8″ switch will be put at 3 1/8″ output of amplifier chain as shown in Fig. 2. One output will be used for input to the next stage amplifier while the other end of the switch (i.e. S3) will be for doing amplifier testing and other prototype RF-plasma interaction experiments. The required switch must have a specified range of return loss, insertion loss and isolation. In this paper, we represent some cases that may affect while designing the RF switch. The analysis in this paper helps to design a low-loss RF switch so that it perfectly matches the transmission line and allows maximum power transfer.

## Critical parameters for RF switch

As the RF switch is made from a coaxial transmission line, its structure and characteristics are similar to a coaxial line. Here in this section, we mention some design criteria and parameters that are very important for the design as described below:

1. Characteristics impedance.
2. Transmission Losses.
3. Isolation.

### Characteristics impedance

The RF switch is of coaxial configuration hence its mechanical design and fabrication are similar to the existing coaxial transmission line. As in the case of the coaxial transmission line, the RF coaxial switch also operates in TEM mode.

The characteristic impedance of the coaxial switch is given by^6^,

1 $${Z}_{0}=\frac{60}{\sqrt{{\epsilon\:}_{r}}}{ln}\frac{b}{a}\,\mathrm{Ohm}$$

Where *ε*_*r*_ is the relative dielectric constant of the media between the inner conductor and outer conductor, “a” is the outer diameter of inner conductor and “b” is the inner diameter of outer conductor. For vacuum or air where *ε*_*r*_ *=* 1, the ration of the radius *b/a* equals 2.3 for an impedance of 50 Ω. In our case switch must be 50 Ohm so that it efficiently matches with 50 Ohm transmission line and dummy load. For an air dielectric (*ε*_*r*_ *=* 1) 50 ohm, *3 *1/8″ coaxial switch, the values of *a* and *b* are 33.4 mm and 76.9 mm respectively.

### Transmission losses

The RF coaxial switch will have two losses – return loss and insertion loss (1), (2).

i. Return loss:

Return loss is the loss of signal power resulting from the reflection caused by a discontinuity in a transmission line. This discontinuity can be a mismatch of the switch with the terminating load or with a device inserted in the line or some obstruction or non-linearity in the line. It is expressed as a ratio in decibels (dB) of incident and reflected power as defined below

2 $$\:RL\left(dB\right)=10lo{g}_{10}\frac{{P}_{in}}{{P}_{ref}}$$

Where, P_*in*_ = incident power and P_*ref*_ = Reflected power.

ii. Insertion loss:

Insertion loss (IL) is the loss of signal power resulting from the insertion of a switch in a transmission line and is expressed in decibels (dB). If the power received at the load end before switch insertion is P_T_ and the power received after insertion of the switch is P_R_, then the insertion loss in dB is given by,

3 $$\:IL\left(dB\right)=10lo{g}_{10}\frac{{P}_{T}}{{P}_{R}}$$

### Isolation

Isolation is an important property of a switch. It prohibits the transmission of power between unconnected ports. It is defined as the ratio of the power between the transmitted port and the unconnected port.

4 $$\:Isolation\left(dB\right)=10{\mathrm{log}}_{10}\frac{{P}_{3}}{{P}_{1}}$$

Where P_1_ is the input power applied at port-1 and P_3_ power measured at port-3.

Where port 1 and port 3 are not connected with each other. In the present RF switch the desired isolation is 40dB or better.

## Design and modeling of two-way coaxial RF switch

**Fig. 3:**
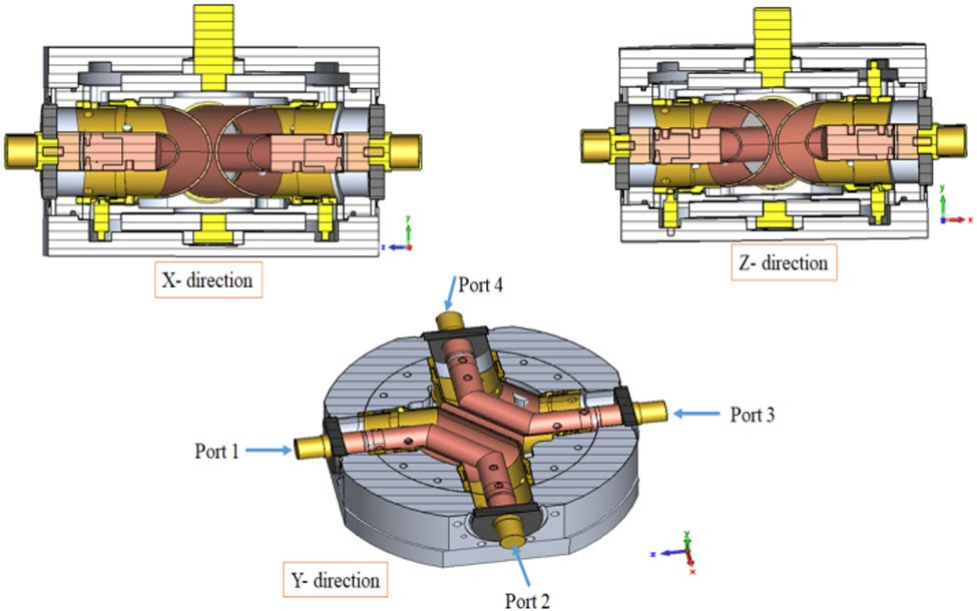
The engineering design layout of coaxial RF switch.

The CAD design model of the co-axial two-way RF switch is shown in Fig. 3. The switch has four ports positioned at exact right angles to each other. Both the inner and outer conductors are configured to connect two ports simultaneously. The switch has a base cover, two sets of inner and outer conductors, a top cover, and a rotating rod that alters the connection between alternative ports. The system is enclosed by an aluminum cover known as the outer RF switch cover. The base and top covers are made of aluminum, while the inner and outer conductors are constructed of copper. The RF connection of all four ports is 3 1/8″ copper coaxial line. The rotating rod’s disc is made up of aluminum, while the outside rod is of brass. To ensure maximum stability and support for the system, the covers are designed with cavities to securely accommodate the conductors. The pairs of conductors are at right angles to each other. This has been achieved by bending the line twice by 45^0^. The bending of 90^0^ has been divided into two step in order to provide a graded transition. Though three step transition would have been further better but it created mechanical fabrication constraints. Small deviations in the dimensions of the inner and outer conductors, bends, and junction regions from their ideal CAD values lead to local impedance mismatches. Even minor dimensional inaccuracies are significant at RF frequencies and directly affect return loss^7^.

**Fig. 4:**
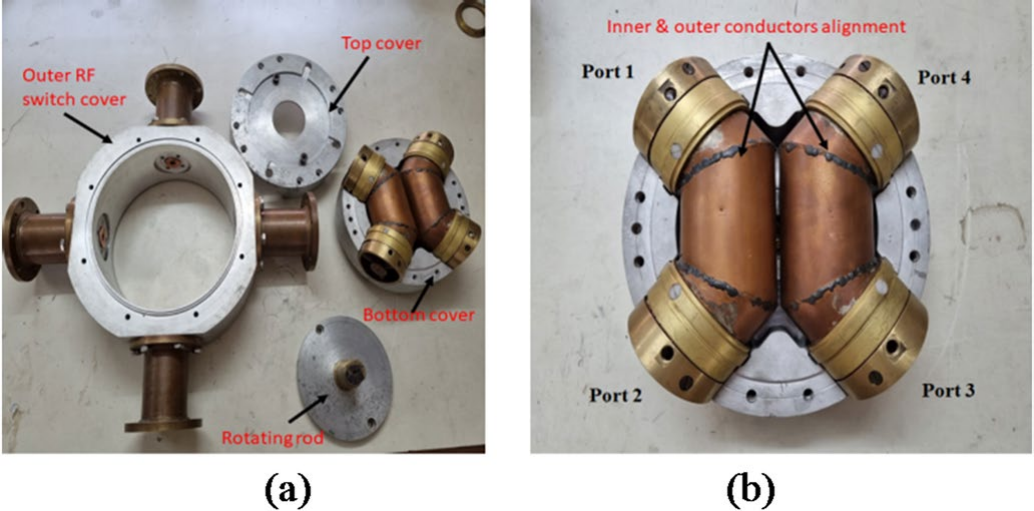
Parts of two-way coaxial RF switch.

The brass shaft on the top side of the switch can rotate the switch in two alternate positions. To transfer the connection to alternate ports, the conductors must not be permanently attached to the ports but should establish connections with them. Finger contacts were utilized for this purpose to establish connections between inner and outer conductors and ports. These fingers contacts are capable of applying sufficient pressure to establish RF connections when needed. Due to the coaxial nature of the system, the inner and outer conductors are aligned co-axially by inserting a Delrin rod between them. Delrin rod has been used as a central conductor support between inner and outer conductor. It has length of 76.9 mm with ± 0.1 mm as manufacturing tolerance with 10.0 mm rod diameter. Delrin rod is used for its mechanical strength and along with acceptable RF properties and can used up to 3 GHz. It has high loss tangent (0.003–0.005) and very good dimensional stability and excellent machinability. Delrin have higher insertion loss and used of Delrin (~ 12.08 cc) is small over the total volume (~ 1717 cc of air) of the RF path so the impact is minimal. Fabricated parts are shown in Fig. 4 below.

## Simulation results and discussion

The two-way coaxial RF switch is modeled and simulated in CST Microwave Studio to verify and improve the design and performance characteristics. The simulation is done for the 3 1/8″ RF switch and the different cases of performance degradation issues. The simulation results are in the form of Return loss (S_11_), Insertion loss (S_21_), and isolation (S_13_) where port 1 and 3 are not connected.

**Fig. 5:**
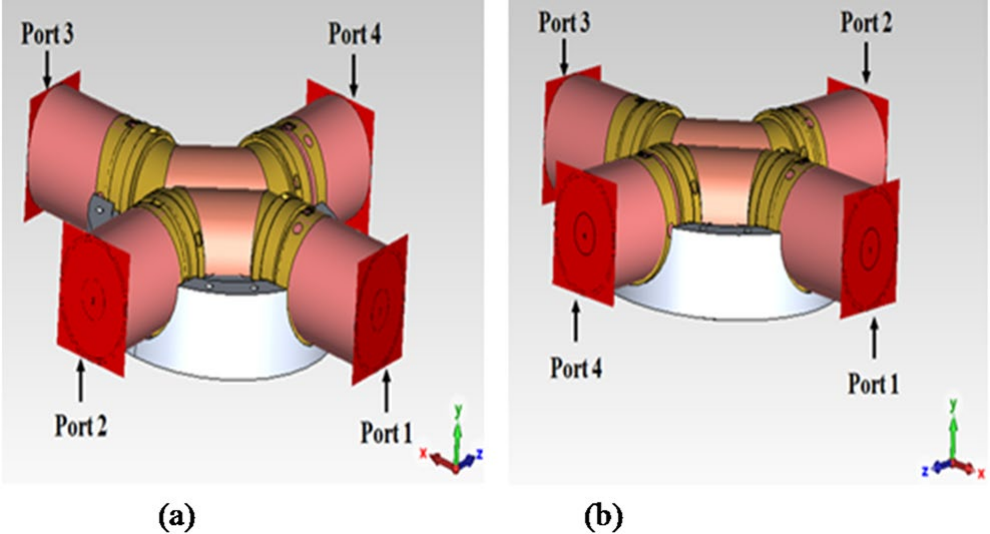
CST model of two-way coaxial RF switch.

The switch has four ports, numbered 1, 2, 3 and 4. In one position of the switch, ports 1 and 2 are connected and ports 3 and 4 are connected. In another situation, ports 1 and 4 are connected and ports 2 and 3 are connected as shown in Fig. 5a, b. Below are the results for the two different connection configurations.

**Fig. 6:**
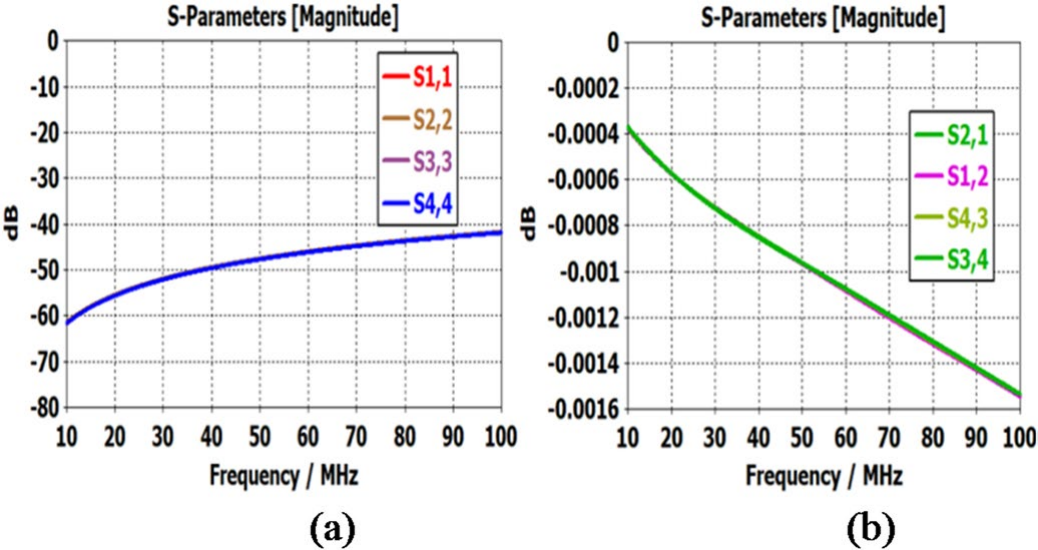
Return loss and Insertion loss of the proposed design for the operating case.

**Fig. 7:**
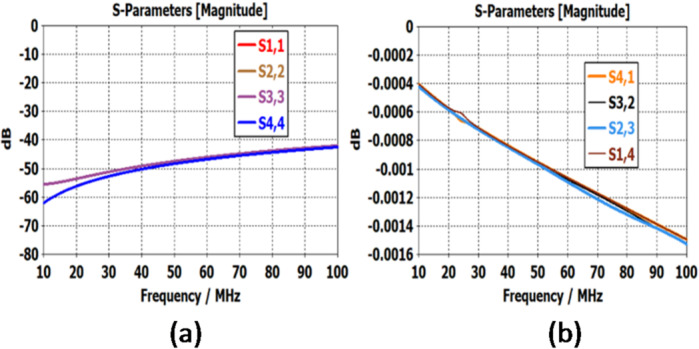
(**a**) Return loss and (**b**) Insertion loss of the proposed design for the operating case.

Case I: Ports 1 and 2 are connected and ports 3 and 4 are connected. Simulation results are plotted in the range of operational frequency 10–100 MHz. Figure 6a shows that the return loss varies from 60.5 to 41 dB while Fig. 6b shows the insertion loss that varies from 0.00039 to 0.0015 dB. These are quite satisfactory results and show the maturity of the design. Case II: Ports 1 and 4 are connected and ports 2 and 3 are connected. Simulation results are plotted in the range of operational frequency 10–100 MHz. Figure 7a shows.

that the return loss varies from 61 to 41 dB while Fig. 7b shows that the insertion loss varies from 0.0004 to 0.00015 dB. These are quite satisfactory results and show the maturity of the design.

**Fig. 8:**
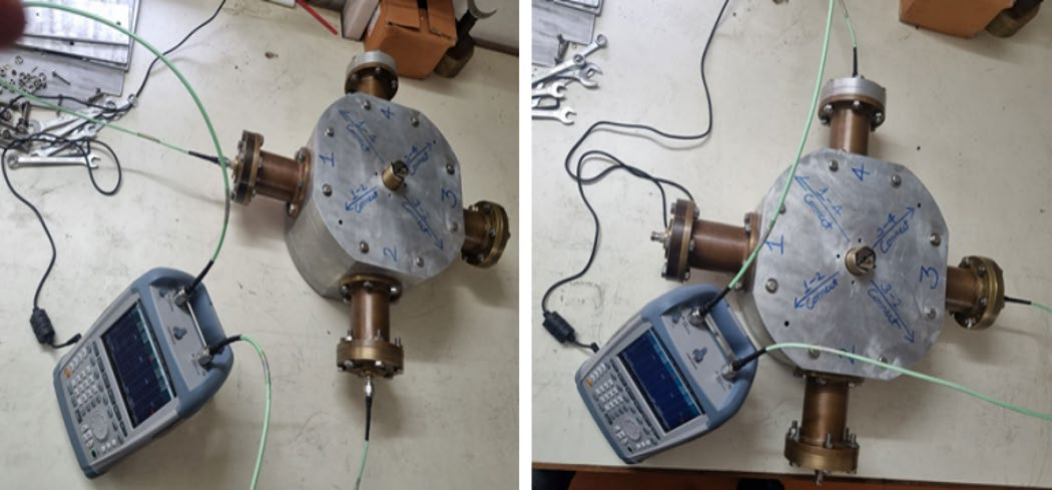
Experimental setup for measurement (case 1).

## Experimental results

### RF switch characteristics (Two Port analysis by VNA)

**Fig. 9:**
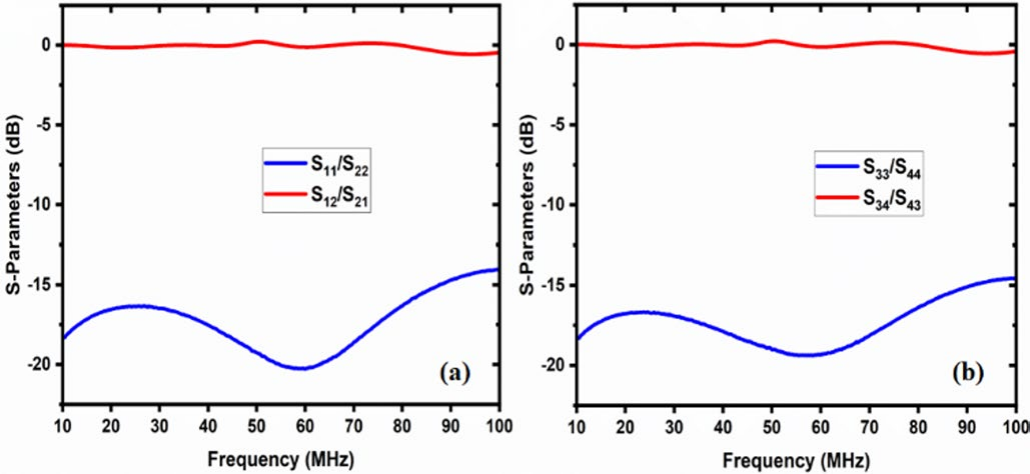
The return loss and insertion loss of case 1 (**a**) connection between port1, port 2, and (**b**) connection between port 3 and port 4.

**Fig. 10:**
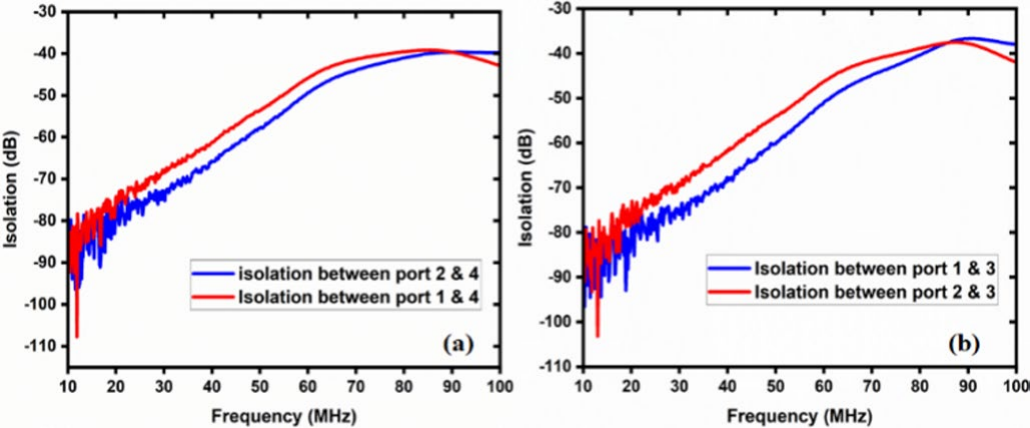
Isolation between unconnected ports in case 1.

The two-way coaxial RF switch is shown Fig. 8 is manufactured indigenously. It is important to substitute the characteristics of the switch that have been measured using a Vector Network Analyzer (Rohde & Schwarz, Model No: FSH8). In the first experimental configuration, port1 to port 2 and port3 to port4 connections are made for measuring reflection or return loss, insertion loss, and isolation. The measured data are tabulated in Table 1 in the operational frequency range of 10–100 MHz. Figure 9 depicts the reflection/return loss (S_11_, and S_22_) that varies from 14.04 dB to 20.27 dB, and the insertion loss (S_21_ and S_12_) that varies from 0.02 to 0.41 dB. The isolation varies from 39 dB to 111 dB as shown in Fig. 10. In the second case, port1 to port 4.

**Fig. 11:**
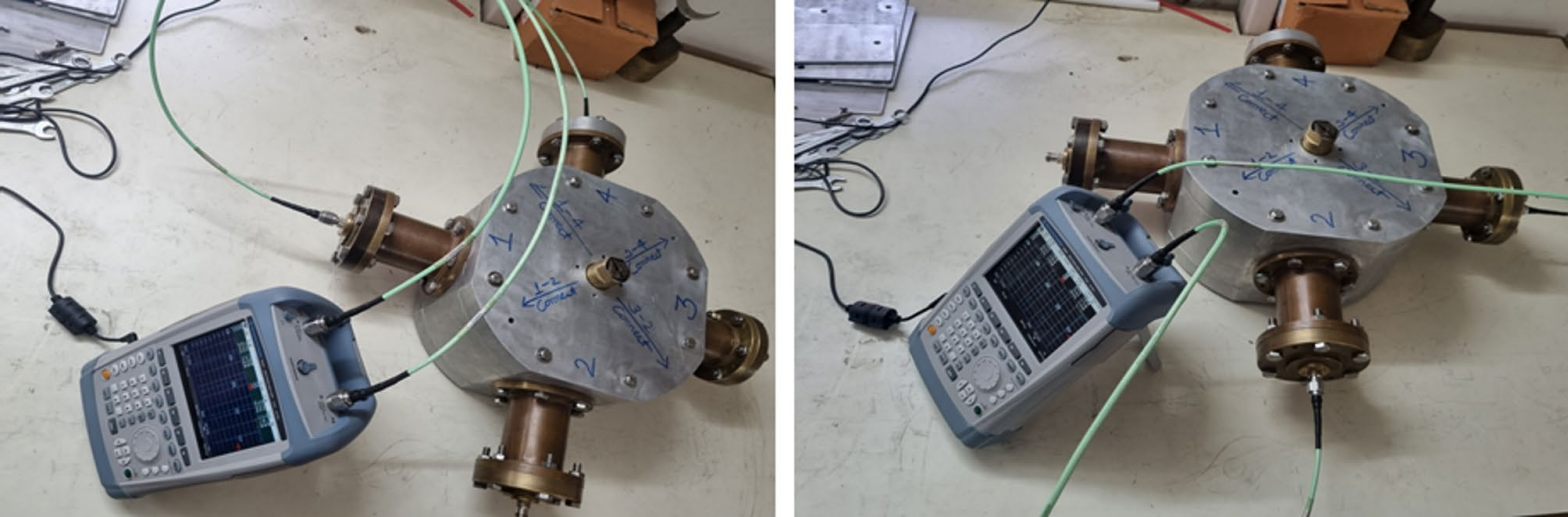
Experimental setup for measurement (case 2).

**Fig. 12:**
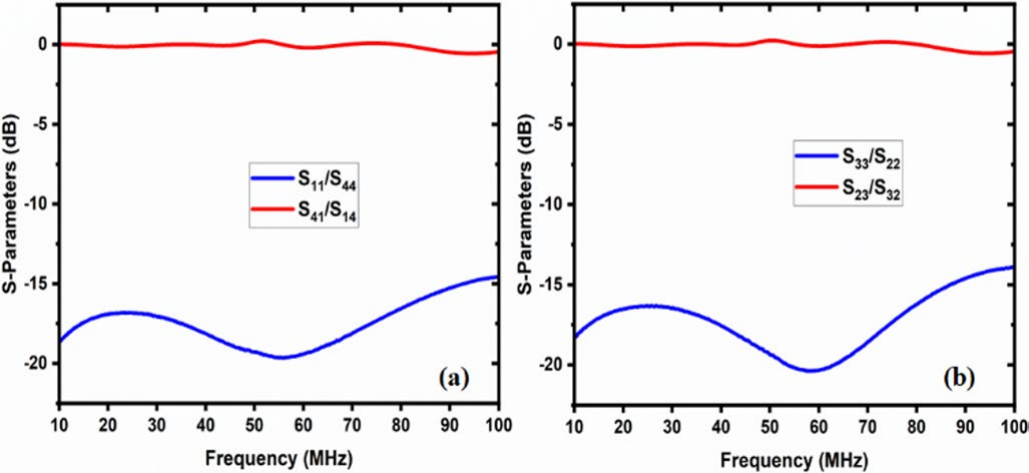
The return loss and insertion loss of case 2 (**a**) connection between port1, port 4, and (**b**) connection between port 2 and port 3.

**Fig. 13:**
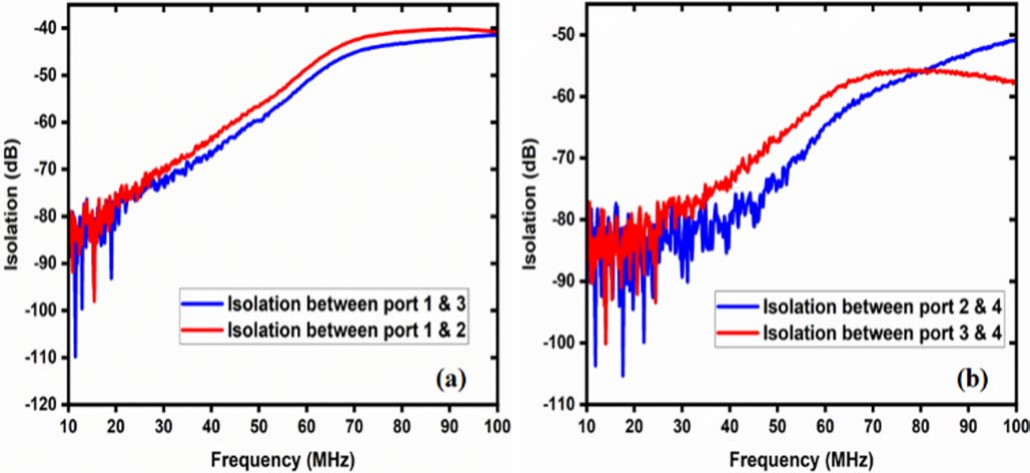
Isolation between unconnected ports in case 2.

In the second case, port1 to port4 and port2 to port3 connections are made (Fig. 11). Figure 12 illustrates the reflection loss (S_33_ and S_44_) that varies from 13.91 dB to 20.33 dB, and the insertion loss (S_41_ and S_32_) that varies from 0.04 to 0.49 dB. The isolation loss is measured between the unconnected ports, and the results are shown in Fig. 13.

**Table 1:** Measured data of two Port analyses by VNA for the operating case.

Frequency (MHz)	Return loss S_11_ (dB)	Insertion Loss S_21_ (dB)	Isolation (dB)	Return loss S_33_ (dB)	Insertion Loss S_43_ (dB)	Isolation (dB)
	Connection between Port 1 to Port 2			Connection between Port 3 to Port 4		
10	18.34	0.02	85.81	18.33	0.02	111.04
20	16.54	0.13	78.00	16.75	0.11	75.23
30	16.44	0.03	73.79	16.89	0.02	69.62
40	17.55	0.03	65.86	17.89	0.02	61.72
50	19.27	0.22	58.02	18.99	0.22	54.33
60	20.27	0.12	49.33	19.31	0.14	46.24
70	18.61	0.11	43.94	18.11	0.09	41.50
80	16.31	0.02	41.07	16.36	0.01	38.88
90	14.70	0.49	39.68	15.09	0.48	37.82
100	14.04	0.44	39.81	14.54	0.41	41.99

The isolation varies from 93.30 to 41.35 dB.

**Fig. 14:**
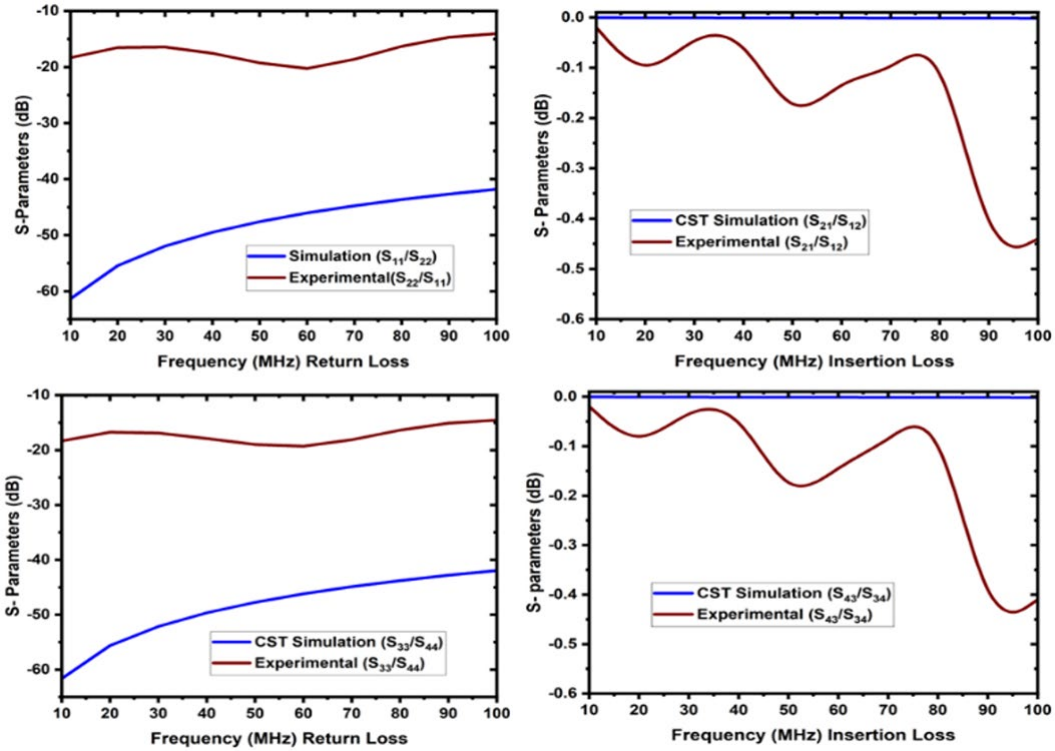
The return loss and insertion loss of case 1 (the connection between port 1 and 2 and port 3, and 4).

**Fig. 15:**
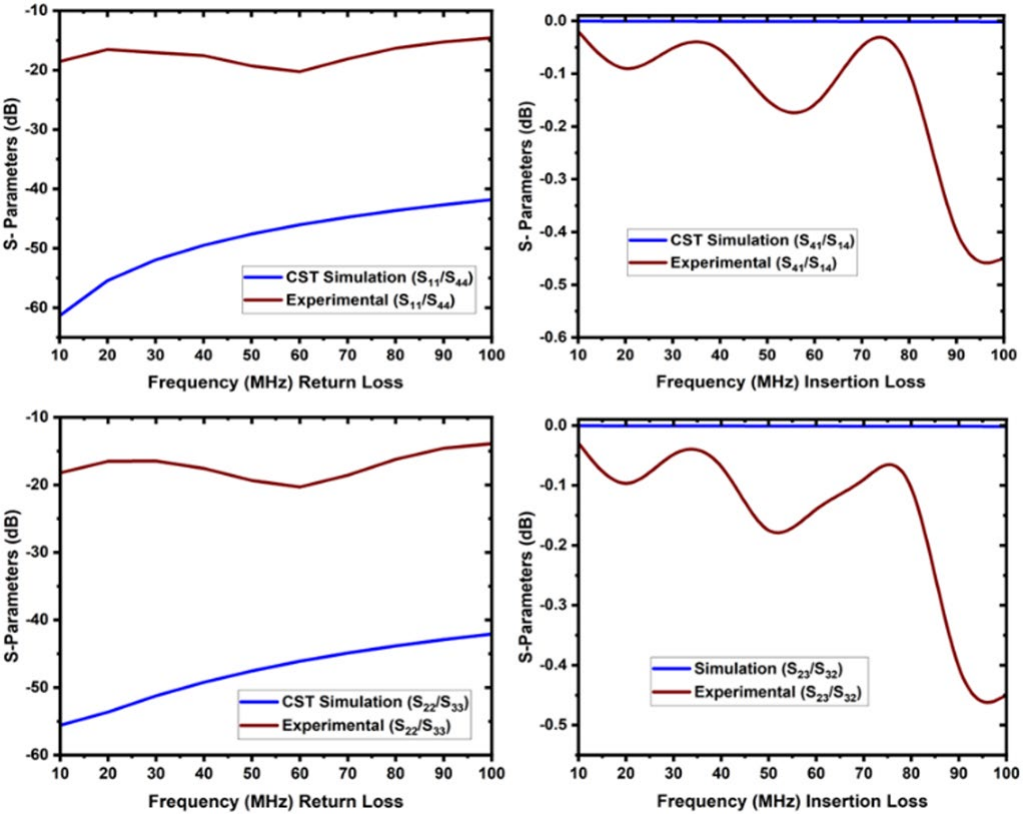
The return loss and insertion loss of case 2 (the connection between port 1, and 4 and port 2 and 3).

**Fig. 16:**
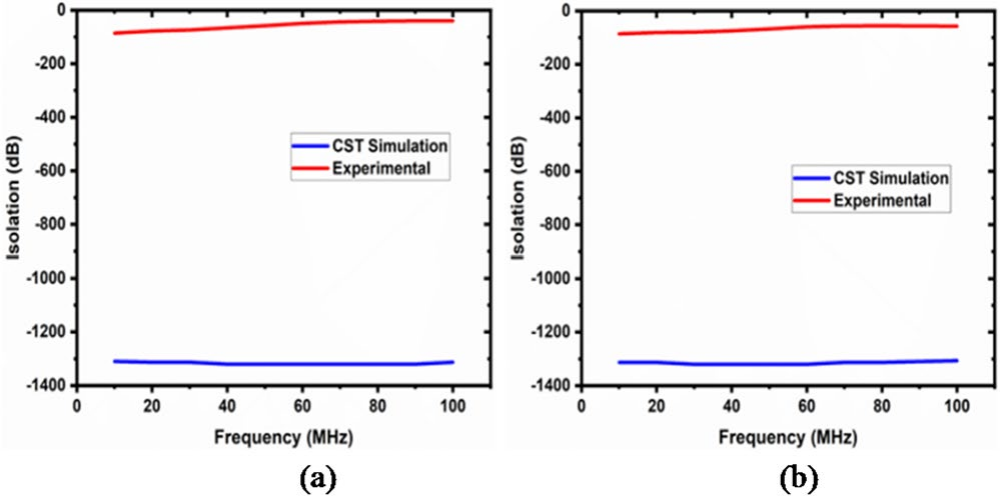
The isolation between unconnected ports in (**a**) case 1 and (**b**) case 2.

The simulation and experimental findings are compared in both cases, as depicted in the figures below. These results are approximately close to each other. Figure 14 displays the return loss and insertion loss in the first case, while Fig. 15 exhibits the return loss and insertion loss in the second case. Figure 16 illustrates the level of isolation between the unconnected ports in both cases (Table 2).

**Table 2:** Measured data of two-port analysis by VNA for the operating case.

Frequency (MHz)	Return loss S_11_ (dB)	Insertion Loss S_41_ (dB)	Isolation (dB)	Return loss S_33_ (dB)	Insertion Loss S_23_ (dB)	Isolation (dB)
	Connection between Port 1 to Port 4			Connection between Port 2 to Port 3		
10	18.56	0.02	86.07	18.22	0.03	93.30
20	16.54	0.12	80.91	16.50	0.13	76.65
30	17.05	0.04	79.06	16.48	0.03	72.99
40	17.55	0.03	74.49	17.57	0.04	66.75
50	19.29	0.17	67.20	19.37	0.22	59.44
60	20.27	0.19	59.90	20.33	0.13	51.25
70	18.12	0.03	56.57	18.56	0.10	45.18
80	16.31	0.02	55.92	16.22	0.01	43.25
90	15.26	0.48	56.70	14.59	0.49	42.28
100	14.57	0.45	57.54	13.91	0.45	41.35

### Low power test (up to 100 W, CW)

**Fig. 17:**
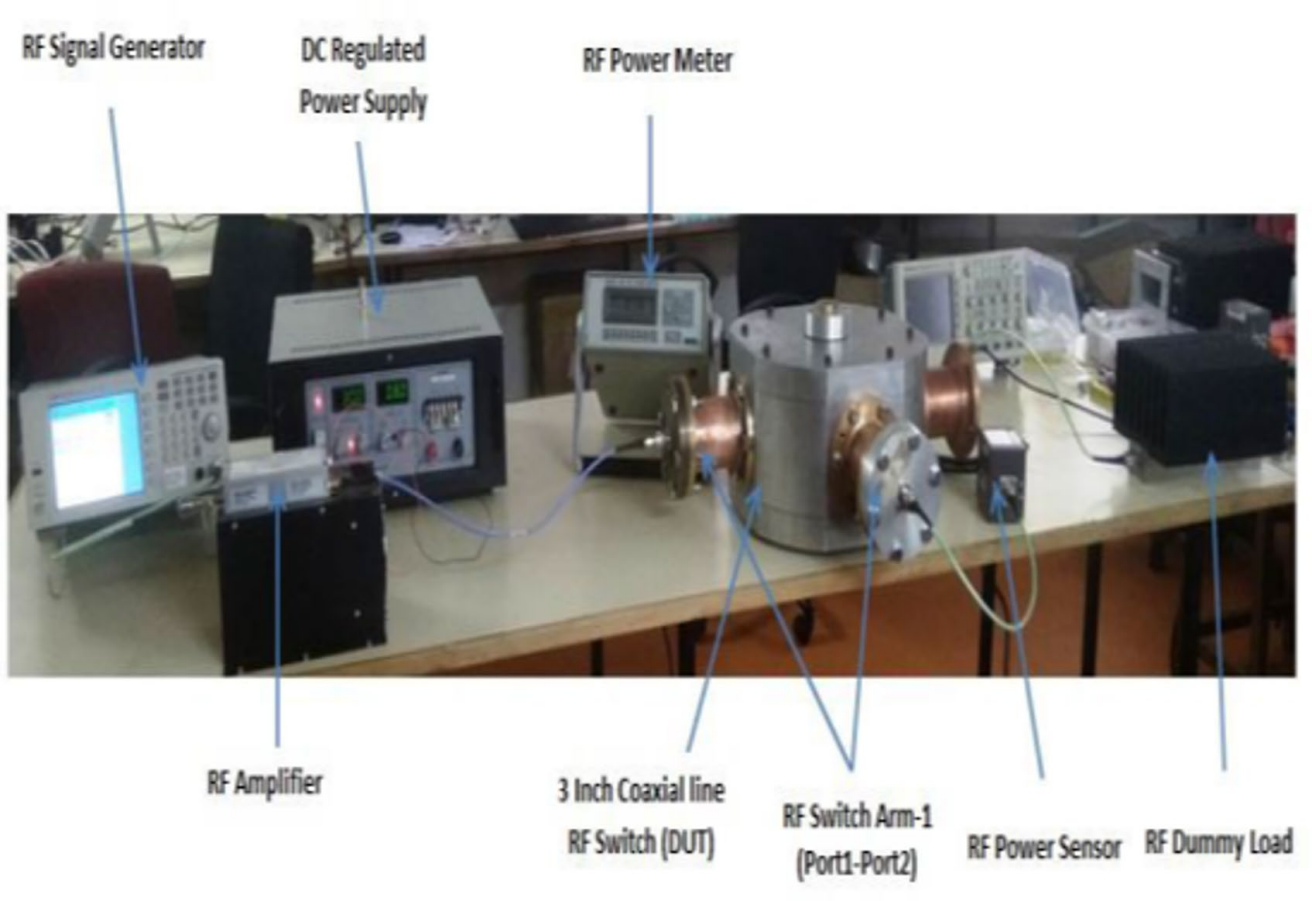
Experimental setup for low power test up to 100 W.

The performance of the two-way coaxial RF switch was evaluated at low power levels, particularly up to 100 W. Figure 17 below illustrates the experimental configuration for the low-power test. Within the experimental setup, an RF power amplifier (Mini-Circuits, USA, Model No: ZHL-100 W-GAN+) was employed to enhance the input signal within the designated frequency range. The input signal to the RF amplifier was provided by a signal generator (Agilent Technologies, Model No: N9310A). A DC-regulated power supply (Sairush Electronic System, Mumbai, Model No: SVL 032030) was utilized to provide the power supply to the RF amplifier and the power output was measured using a power meter (Bird Electronic Incorp, USA, Model No: 4421).

The input signal generator was connected to the RF power amplifier for amplification. The amplified signal was transmitted from the RF power amplifier to port 1 of the RF switch via the cable, while port 2 was connected to the power meter to measure the output power. The collected data has been arranged and presented in the Table 3 below. During the low-power test, the measured reflected power ranged from 0.01 to 0.2 Watts, while the measured VSWR ranged from 1.04 to 1.09 within the frequency range of 20–100 MHz.

**Table 3:** Measured experimental data for low-power test setup.

Frequency (MHz)	Input Power (W)	Reflected Power (W)	SWR	Input Power (W)	Reflected Power (W)	SWR
	Connection between Port 1 and Port 2			Connection between Port 3 and Port 4		
20	25.90	0.0136	1.046	26.29	0.0135	1.048
	43.54	0.0233	1.047	43.52	0.0235	1.047
	66.68	0.0370	1.048	66.70	0.0389	1.048
	82.14	0.0671	1.058	83.02	0.0669	1.058
	100.54	0.207	1.094	100.85	0.2072	1.095
40	23.46	0.0230	1.064	23.49	0.024	1.067
	35.47	0.0355	1.062	38.98	0.039	1.067
	61.56	0.0571	1.063	67.73	0.062	1.065
	82.95	0.070	1.059	84.92	0.078	1.063
	102.58	0.0799	1.057	100.29	0.087	1.060
60	23.45	0.039	1.085	23.56	0.042	1.089
	39.82	0.0625	1.084	40.04	0.070	1.087
	60.62	0.090	1.080	60.71	0.098	1.084
	79.45	0.109	1.077	80.09	0.118	1.080
	100.99	0.1213	1.072	101.65	0.1362	1.076
**80**	23.32	0.044	1.091	23.46	0.048	1.095
	39.25	0.0711	1.089	39.40	0.076	1.092
	60.02	0.0970	1.084	60.39	0.1035	1.086
	79.75	0.117	1.079	79.66	0.128	1.083
	101.25	0.1355	1.076	100.38	0.150	1.080
100	23.85	0.0409	1.086	23.99	0.043	1.088
	38.75	0.0652	1.085	39.25	0.068	1.087
	58.06	0.0872	1.081	58.46	0.091	1.082
	78.10	0.106	1.076	77.63	0.111	1.074
	101.32	0.130	1.074	100.57	0.137	1.076

## Conclusion

The design, simulation, and characterization of this 3−1/8-inch coaxial two-way RF switch represent a significant convenience for RF-Plasma discharge experiments. It will help to utilize the full potential of stage-wise development of the RF generator. As RF generation is a very costly affair, this switch makes it possible to use the same amplifier for more than one purpose without making significant and costly changes to the transmission line layout. Making layout changes using bends etc. for RF power diversion for different experiments with single multi-stage RF generators in large-size rigid coaxial transmission lines is very uneconomical and slow. The developed switch is a very economical, fast, and convenient alternative.

## Data Availability

All the data generated during the present investigation are presented within the manuscript.
